# Obesity and Uncontrolled Diabetes Predict Depression in HF Patients

**DOI:** 10.3390/jcm10235663

**Published:** 2021-11-30

**Authors:** Albenita Fetahu, Kaltrinë Rrustemi, Michael Y. Henein, Besim Bytyçi, Flamure Mehmeti, Ibadete Bytyçi, Lulzim Kamberi

**Affiliations:** 1 Department of Nursing, Universi College, 10000 Prishtina, Kosovo; albenita.fetahu1@gmail.com (A.F.); kaltrinna_rrustemi@hotmail.com (K.R.); i.bytyci@hotmail.com (I.B.); 2 Institute of Public Health and Clinical Medicine, Umea University, 90187 Umea, Sweden; michael.henein@umu.se; 3 Molecular and Clinic Research Institute, St George University, London SW17 OQT, UK; 4 Institute of Fluid Dynamics, Brunel University, London UB8 3PH, UK; 5 Clinic of Rheumatology, University Clinical Centre of Kosovo, 10000 Prishtina, Kosovo; besim.bytyqi@hotmail.com; 6 Clinic of Cardiology, University Clinical Centre of Kosovo, 10000 Prishtina, Kosovo; flamuremehmeti@gmail.com

**Keywords:** heart failure, HFpEF, HFrEF, depression scale

## Abstract

Background and aim: Heart failure (HF) is a clinical syndrome associated with poor quality of life and prognosis, and premature mortality. The aim of this study was to assess the prevalence of depression and its risk factors in HF patients. Methods: The study included 151 HF patients (mean age of 66.6 ± 11 years, 52.3% female). Based on ejection fraction (EF), the study cohort was divided into the following two groups: group-I: HFpEF patients (EF ≥ 50%, *n* = 47) and group-II: HFrEF patients (EF < 40%, *n* = 104). For the enrolled patients, demographic, clinic and echocardiographic indices, and depression scale results were collected. Results: The patients with HF and depression were older, mostly females, more obese, and had a higher glycemic level and higher NYHA functional class compared with the patients without depression (*p* < 0.05 for all). The left ventricle (LV) and left atrial (LA) dimensions were larger, and EF was lower, in patients with depression compared to those without depression (*p* < 0.05 for all), while the right ventricle (RV) measurements did not differ (*p* > 0.05). The same parameters remained significantly different when the patients were divided into HFpEF and HFrEF. The depression scale correlated with glycemic level (r = 0.51, *p* = 0.01), obesity (rpb = 0.53, *p* = 0.001), age (r = 0.47, *p* = 0.02), and severity of NYHA class (rpb = 0.54, *p* = 0.001). On a multivariate model, BMI ≥ 30 kg/m^2^, OR 1.890 (1.199 to 3.551; 0.02) glycemic level ≥ 8.5 mmol/L, OR 2.802 (1.709 to 5.077; *p* = 0.01), and NYHA class > 2, OR 2.103 (1.389 to 4.700; *p* = 0.01), proved to be the most powerful independent predictors of depression, in the group as a whole. Obesity and uncontrolled diabetes predicted depression, irrespective of EF. Conclusions: In this modest cohort of HF patients, obesity and uncontrolled diabetes were independent predictors of depression, irrespective of LV systolic function. This emphasizes the important role of medical education for better control of such risk factors.

## 1. Introduction

Heart failure (HF) is a known clinical syndrome and constitutes a major public health problem associated with poor quality of life and prognosis, and premature mortality [1]. It is well established that depression is an independent risk factor for HF due to coronary artery disease and other cardiac diseases [2], and symptoms of depression are also associated with a greater risk of adverse cardiac events [3]. It has been suggested that HF itself could have a causal relationship with the onset of depression, since some parts of the brain are especially vulnerable to suboptimal perfusion, especially with reduced cardiac output and underlying cerebral atherosclerosis [4]. The classical hypothesis supports a link between the onset of depression and suffering from chronic stress in patients with chronic heart disease [5]. In addition, depression itself reduces the quality of life, not only in isolated psychiatric disorders, as a primary condition, but also in other concomitant diseases. Despite the fact that depression should be approached within a care program in patients with HF, it is not always considered a serious issue, and clinicians may only focus on the heart problem, thus underestimating the clinical impact of depression [6,7].

The aim of this study was to assess the prevalence of depression among HF patients and to identify the risk factors predicting it.

## 2. Methods

### 2.1. Study Population

We studied 151 consecutive patients with clinical signs and symptoms of HF with New York Heart Association (NYHA) functional class I–IV, according to the current ESC guidelines [8]. Based on left ventricular ejection fraction (EF), patients were divided into HF with reduced EF (EF < 40%, HFrEF, *n* = 104) and HF with preserved EF (EF ≥ 50%, HFpEF, *n* = 47; Figure S1). All patients were referred to the Clinic of Cardiology, University Clinical Center of Kosovo, Prishtina, Kosovo between June and October 2019. Exclusion criteria were as follows: history of congenital heart disease, pacemaker implantation, valvular surgery, cardiac transplantation, chronic obstructive pulmonary disease (COPD) or recent acute coronary syndrome, stroke, age < 18 years and patients with psychiatric disorders diagnosed before heart failure. The study was approved by the local institutional review board and all patients gave written informed consent before enrollment in the study.

### 2.2. Data Collection

Detailed history, clinical, ECG and echocardiographic assessments were obtained in all patients. Cardiac risk factors, such as diabetes mellitus (DM), arterial hypertension (AH), dyslipidemia, coronary heart disease (CHD), and smoking, were assessed. The routine biochemical test results were collected, including complete blood count, blood glucose, cholesterol, triglyceride, albumin, protein, kidney and liver function as well as weight and height measurements. According to the World Health Organization conventional international risk factor assessment and cut-off values for body mass index (BMI), underweight was defined as BMI < 18.5 kg/m^2^, normal weight was BMI 18.5–24.9 kg/m^2^, overweight was defined as BMI of 25–29.9 kg/m^2^, and obesity as BMI ≥ 30 kg/m^2^ [9]. Echocardiographic indices, such as left ventricle end diastolic dimension (LVEDD), LV systolic dimension (LVESD), LV ejection fraction (LVEF), and left atrial (LA), right atrial (RA) and right ventricle (RV) dimensions, were also collected.

### 2.3. Depression Assessment

Depression was assessed using Patient Health Questionnaire-9 (PHQ-9), an instrument for screening, diagnosing, monitoring and measuring the severity of depression. PHQ-9 score obtained by adding score for each question with score that ranged 0–27. Total scores of 5, 10, 15, and 20 represent cut-off points for mild, moderate, moderately severe and severe depression, respectively. A score of PHQ-9 ≥ 10 was used to define the presence of moderate depression [10].

### 2.4. Statistical Analysis

Statistical analysis was performed using SPSS Software Package version 26.0 (IBM Corp., Armonk, NY, USA). Data are summarized using frequencies (percentages) for categorical variables and mean ± standard deviation (SD) for continuous variables or median interquartile (IRQ) ranges. Continuous data were compared with two-tailed Student *t* test and discrete data with chi-square test. The degrees of association between clinical, biochemical and depression scales were determined using the Pearson’s correlation coefficient, in the case of continuous variables, chi-square test (categorical variables) and point biserial correlation in the case of categorical and continuous variables. A significant difference was defined as *p* value < 0.05 (2-tailed). 

## 3. Results

### 3.1. Demographic and Clinical Data of HF Patients 

One hundred and fifty-one patients were included in the study, with a mean LVEF of 47 ± 7.8% and NYHA functional class of 2.3 ± 1.0. The patients’ mean age was 66.6 ± 11 years, and 52.3% were females and 19.2% were current smokers. Further, 28.5% of the patients were obese, 66.2% were hypertensives (AH), 48.4% were diabetics (DM), 43.8% had coronary heart disease (CHD), and 47.1% had atrial fibrillation (AF). Additionally, 78.2% of the patients were receiving aspirin, 82.8% ACE/ARBs, 70.9% diuretics, 73.5% beta blockers, 15.2% calcium channels blockers, and 17.9% were undergoing anticoagulation therapy (Table S1). The biochemical and echocardiographic data are presented in Tables S2 and S3.

### 3.2. Demographic and Clinical Data of the Patients with and without Depression

Out of the 151 studied patients, 33 (19.8%) had a total score > 10 or were diagnosed with moderate depression. The patients with HF and depression were older (*p* = 0.01), mostly females (*p* = 0.02), more obese (*p* = 0.02), and had a higher NYHA functional class (*p* = 0.01) compared to those without depression. AF and DM were more prevalent among HF patients with depression (*p* < 0.05 for both), while AH, dyslipidemia, and CHD were not significantly different between the groups (*p* > 0.05 for all). Similarly, the HF patients with depression were receiving more anticoagulants and diuretics (*p* < 0.05 for both) compared to those without depression, but the other medications were not different between the groups (*p* > 0.05 for all; Table S1). 

### 3.3. Biochemical and Echocardiographic Indices of Patients with and without Depression

The HF patients with depression had a higher glycemic level, higher markers of kidney dysfunction, and lower iron levels (*p* < 0.05 for all) compared to those without depression. The other biochemical indices did not differ between the groups (*p* > 0.05 for all; Table S2). Additionally, the HF patients with depression had larger LVEDD (*p* = 0.03), larger LVESD (*p* = 0.02), lower LVEF (*p* = 0.01), and a larger LA dimension (*p* = 0.03) compared to those without depression. The RA and RV measurements did not differ between the two patient groups (Table S3).

### 3.4. Demographic and Clinical Data of Patients according to EF

The HFrEF patients with depression were predominantly female (*p* = 0.03) and had a higher NYHA functional class (*p* = 0.02) compared to those without depression. DM, obesity, and AF were more prevalent among the HFrEF patients with depression (*p* < 0.05 for all), while AH, dyslipidemia, and CHD were not different from those without depression (*p* > 0.05). Although the HFrEF patients with depression were receiving more diuretics and anticoagulants, the other medications were not different from those without depression. Among the HFpEF patients, the depression group were older compared to those without depression (*p* = 0.02). Similarly to the HFrEF group, the frequency of females, obesity, DM, and AF were higher in the HFpEF patients with depression compared to those without depression. In contrast, the frequency of patients receiving anticoagulation medications was higher, and antiarrhythmic drugs tended to be more prevalent among the group with depression compared to the group without depression (Table 1). 

### 3.5. Biochemical and Echocardiographic Indices of Patients according to EF

In the HFrEF patients, the glycemic level and markers of kidney dysfunction were higher, and the iron level was lower in patients with depression compared to those without depression (*p* < 0.05 for all), but the other biochemical indices were not different (*p* > 0.05). The echocardiographic indices, including the LV systolic and diastolic dimensions and EF, as well as the LA, RA, and RV dimensions, did not differ between the groups (*p* > 0.05 for all). Almost similar results were shown in the HFpEF groups (*p* > 0.05 for all; Table 2 and Table 3).

### 3.6. Correlation between Cardiovascular Risk Factors and Depression

**Figure 1:**
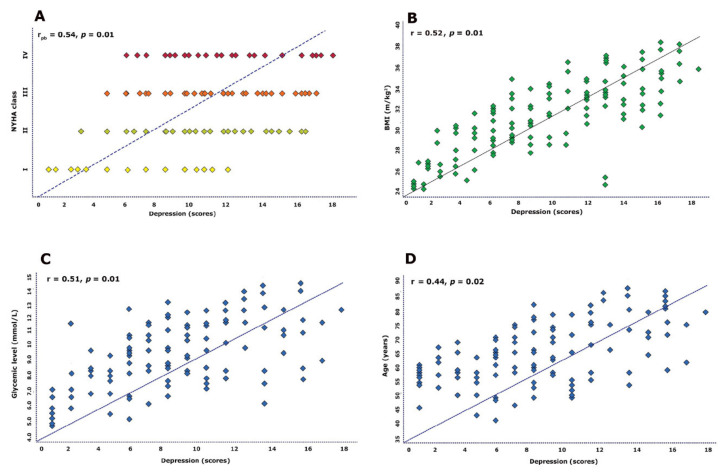
Correlation of demographic and clinical indices with depression; (**A**) correlation between New York Heart Association class (NYHA) class and depression; (**B**) correlation between Body mass index (BMI) and depression; (**C**) correlation between glycemic level and depression; (**D**) correlation between age and depression.

The depression scale correlated with the glycemic level (r = 0.51, *p* = 0.01), BMI (r = 0.52, *p* = 0.01), severity of NYHA class (r_pb_ = 0. 54, *p* = 0.001), and only modestly with age (r = 0.43, *p* = 0.02; Figure 1). In contrast, no relationship was found between depression and AH (r = 22, *p* = 0.11) or dyslipidemia (r = 0.17, *p* = 0.31), but AF tended to have a relationship with depression (r_pb_ = 0.21, *p* = 0.08). 

### 3.7. Predictors of Depression in the Studied Cohort 

The prevalence of depression in the study participants was 21.8%, with no difference between HFrEF and HFpEF (22.1% vs. 21.2%, respectively; *p* = ns). In the univariate analysis, age (*p* = 0.03), female gender (*p* = 0.04), NYHA class >II (*p* = 0.01), BMI ≥ 30 kg/m^2^ (*p* = 0.01), and glycemic level ≥ 8.5 mmol/L (*p* = 0.001) predicted moderate depression. In the multivariate model, BMI ≥ 30kg/m^2^, OR 1.890 [(1.199 to 3.551) 0.02], glycemic level ≥ 8.5 mmol/L, OR 2.802 [(1.709 to 5.077), *p* = 0.01], and NYHA class > 2, OR 2.103 [(1.389 to 4.700) *p*=0.01], proved to be the most powerful independent predictors of depression in the HF cohort (Table 4) as a whole. Testing different factors that predict depression, based on LV EF, showed that NYHA class >II (OR 2.091; *p* = 0.01), obesity (OR 1.911; *p* = 0.03), and uncontrolled DM (OR 2.013; *p* = 0.01) were independent predictors of depression in HFrEF patients, while female gender (OR 1.591; *p* = 0.04), obesity (OR 1.926; *p* = 0.02), and uncontrolled diabetes (OR 1.703; *p* = 0.03) were independent predictors of depression in the HFpEF group (Table 4). Collinearity between these measurements was not met based on VIF < 10 for all the predictors.

## 4. Discussion

### 4.1. Findings

Almost 22% of our studied patients with HF had moderate depression, irrespective of left ventricular EF. Moderate signs and symptoms of HF, based on NYHA, obesity, and uncontrolled diabetes, predicted the occurrence of depression in the group of patients as a whole. Obesity, uncontrolled diabetes, and NYHA class were independent predictors of depression in the HFrEF patients, while female gender, obesity, and uncontrolled diabetes were the respective predictors in the HFpEF group.

### 4.2. Data Interpretation

The overall prevalence rate of depression in our modest cohort of HF patients was 21.8%, suggesting a clinical problem of similar impact to that previously reported in CHD patients [11,12]. Moderate depression was found in at least 20% of the patients, and such evidence cannot be clinically ignored, since it supports former reports that showed a relationship between HF and depression [13,14,15]. In this study, we also compared the prevalence of depression accordingly to left ventricular EF, in HFrEF and HFpEF patients. A moderate NYHA class, uncontrolled diabetes, and obesity were the main independent predictors of depression in the HF cohort as a whole. This finding is supported by previous reports, which showed a significant correlation between NYHA class and depression, with the prevalence of depression increasing with a higher NYHA functional class [16,17,18]. When splitting the patients’ cohort, according to LV EF, into reduced (HFrEF) and preserved (HFpEF), our analysis showed some similarities and some differences in the risk factors predicting depression. Uncontrolled diabetes and obesity predicted the depression scale, irrespectively of EF. Again, this finding supports previous reports that showed patients with type 2 diabetes being at high risk for cardiovascular disease, heart failure, and depression [19]. Recognition of this common finding should have significant clinical implications, with optimum management of depression resulting in improved glycemic control [20]. Another related risk factor that we identified to predict depression, irrespective of LVEF, was obesity. This finding is supported by a large population study that showed obesity as an important factor associated with depression and HF [21]. It is well known that obesity, metabolic syndrome, and diabetes are three stages of the same disturbed pathophysiology. Our findings that showed uncontrolled diabetes and obesity as independent predictors of depression are then expected in a cohort of HF patients. Differences between the groups, according to LV EF, were of some interest. Female gender was proved to be an independent predictor of depression in HFpEF, but not in HFrEF. This finding can be explained by the higher frequency of female patients with HFpEF compared to HFrEF, which is expected based on the morphological presentation of the disease [22]. The stronger predictive value of the NYHA class of depression in HFrEF, but not HFpEF, could be explained by the higher incidence of CAD in the former compared with the latter [14,23].

### 4.3. Clinical Implications

Clinicians need to focus more on the management of obesity, uncontrolled diabetes, and depressive symptoms in HF patients. Nonetheless, the findings of this study provide important information about some modifiable risk factors, including obesity and uncontrolled diabetes, that contribute to the occurrence of depression. If these factors are ignored, they can only lead to worsening of the overall clinical condition and quality of life of HF patients.

### 4.4. Limitations

This study has some limitations. The relatively small number of participants reflects the difficult nature of the study, design, and data collection. Defining depression on the basis of symptom severity, depending on questionnaires rather than a diagnostic interview, could be inadequate in some cases. The exact duration of the risk factors was not available, hence their accurate impact could be somewhat limited. In addition, the assessment of natriuretic peptides could have also helped to better predict the prognosis and severity of the disease, but this facility was not available. 

## 5. Conclusions

In this modest cohort of HF patients, obesity, uncontrolled diabetes, and severity of NYHA functional class were independent predictors of depression. This emphasizes that the symptoms of depression should be considered in HF patients, and routine screening for depression and anxiety should be of crucial importance in high-risk patients.

## Figures and Tables

**Table 1:** Demographic and clinical data between HF patients with and without depression according to EF.

Variable	HFrEF without Depression (*n* = 81)	HFrEF with Depression (*n* = 23)	*p* Value	HFpEF without Depression (*n* = 37)	HFpEF with Depression (*n* = 10)	*p* Value
Clinical indices						
Age	71.1 ± 11	68.2 ± 10	0.12	60.1 ± 12	68.2 ± 11	0.04
Sex (female, %)	41 (50.6)	14 (60.8)	0.03	18 (48.6)	6 (60)	0.01
Smoking (*n*, %)	21 (25.9)	5 (21.7)	0.12	8 (21.6)	2 (20)	0.33
BMI (m/kg^2^)	26.3 ± 3.5	30.4 ± 2.9	0.04	27.9 ± 3.3	28.8 ± 4.4	0.13
Underweight (*n*, %)	1 (1.23)	0 (0)	0.04	0 (0)	0 (0)	0.77
Normal weight (*n*, %)	29 (35.8)	11 (47.8)	0.03	13 (43.2)	4 (40)	0.31
Overweight (*n*, %)	29 (35.8)	10 (43.5)	0.04	15 (40.5)	5 (50)	0.003
Obese (*n*, %)	21 (25.9)	8 (34.7)	0.02	10 (27.0)	4 (40)	0.002
SBP (mmHg)	106 ± 15	108 ± 14	0.63	111 ± 16	109 ± 14	0.70
DBP (mmHg)	78.1 ± 10	75 ± 13	0.32	83 ± 12	78 ± 16	0.52
HR (beats/min)	75 ± 10	73 ± 11	0.31	76 ± 13	74 ± 10	0.41
NYHA class	1.8 ± 1.0	2.9 ± 1.1	0.02	1.8 ± 0.9	2.9 ± 1.1	0.03
**Comorbidities**						
AH (*n*, %)	56 (69.1)	14 (60.9)	0.08	24 (64.8)	6 (60)	0.10
DM (*n*, %)	39 (48.1)	13 (56.5)	0.04	16 (43.1)	5 (50)	0.03
Dyslipidemia	23 (28.4)	7 (30.4)	0.51	10 (27.0)	3 (30)	0.61
CHD (*n*, %)	41 (50.6)	12 (52.2)	0.21	14 (37.8)	4 (40)	0.33
AF (*n*, %)	40 (51.8)	15 (65.2)	0.01	12 (32.4)	4 (40)	0.03
**Drugs**						
Aspirin (*n*, %)	64 (79.1)	18 (78.2)	0.27	28 (75.7)	8 (80)	0.47
ACE/ARBs (*n*, %)	67 (82.7)	19 (82.6)	0.55	30 (81.1)	8 (80)	0.35
Diuretics (*n*, %)	58 (71.6)	20 (86.9)	0.001	22 (59.5)	7 (70)	0.02
BB (*n*, %)	64 (79.1)	19 (78.2)	0.20	26 (70.3)	7 (70)	0.80
CCB (*n*, %)	11 (13.6)	3 (13.1)	0.51	7 (18.9)	2 (20)	0.33
Antiarrhythmic (*n*, %)	14 (17.3)	5 (21.7)	0.21	6 (16.2)	2 (20)	0.09
Anticoagulation (*n*, %)	15 (18.5)	7 (30.4)	0.01	4 (10.8)	1 (10)	0.21
Statins (*n*, %)	29 (29.6)	7 (30.4)	0.41	6 (16.2)	1 (10)	0.11

Body mass index (BMI), New York Heart Association (NYHA), arterial hypertension (AH), diabetes mellitus (DM), dyslipidemia, coronary heart disease (CHD), atrial fibrillation (AF); Systolic blood pressure (SBP), diastolic blood pressure (DBP), heart rate (HR), angiotensin converting enzyme (ACE), angiotensin II receptors blockers (ARBs), beta clockers (BB), channel calcium blockers (CCB).

**Table 2:** Laboratory data between HF patients with and without depression according to EF.

Variable	HFrEF without Depression (*n* = 81)	HFrEF with Depression (*n* = 23)	*p* Value	HFpEF without Depression (*n* = 37)	HFpEF with Depression (*n* = 10)	*p* Value
Laboratory data						
Glucose (mmol/L)	5.9 ± 2.2	5.6 ± 1.2	0.02	5.6 ± 1.2	8.2 ± 3.2	0.02
Urea (mmol/L)	9.4 ± 5.6	16.1 ± 6.2	0.03	8.3 ± 4.2	14 ± 5.1	0.04
Creatinine (umol/L)	125 ± 11	165 ± 25	0.01	123 ± 11	145 ± 19	0.02
Bilirubin (mg/dL)	4.1 ± 1.1	5.5 ± 1.1	0.21	3.8 ± 1.3	4.9 ± 1.3	0.33
ALT (U/L)	30 ± 10	37 ± 11	0.32	34 ± 11	30 ± 10	0.18
AST (U/L)	33 ± 13	28 ± 10	0.51	30 ± 10	28 ± 14	0.31
Albumin (g/L)	30.3 ± 8.5	33 ± 9.1	0.24	31 ± 10	33 ± 9.6	0.32
Protein (g/L)	59 ± 11	61 ± 10	0.24	62 ± 10	64 ± 11	0.28
Cholesterol (mmol/L)	6.8± 3.2	6.2 ± 3.1	0.22	5.9 ± 3.5	6.8 ± 3.1	0.38
Triglyceride (mmol/L)	1.9 ± 0.8	2.1 ± 0.9	0.31	1.9 ± 0.8	2.2 ± 0.7	0.22
WBC (10^3^/mm^3^)	9.4 ± 4.3	8.2 ± 4.6	0.29	9.2 ± 4.6	8.9 ± 3.4	0.37
RBC (10^6^/mm^3^)	3.9 ± 1.2	4.4 ± 1.2	0.23	4.1 ± 1.2	4.7 ± 1.1	0.38
Platelet (10^3^/mm^3^	212± 23	217± 22	0.21	209± 21	215 ± 25	0.11
Iron (umol/L)	8.8 ± 2.8	13.1 ± 3.1	0.03	10.2 ± 3.1	9.9 ± 2.5	0.20

ALT: alanine aminotransferase; AST: aspartate aminotransferase; RBC: red blood cell; WBC: white blood cell.

**Table 3:** Echocardiographic data between HF patients with and without depression according to EF.

Variable	HFrEF without Depression (*n* = 81)	HFrEF with Depression (*n* = 23)	*p* Value	HFpEF without Depression (*n* = 37)	HFpEF with Depression (*n* = 10)	*p* Value
LV EDD (cm)	5.7 ± 0.6	6.1 ± 0.7	0.10	5.1 ± 0.4	5.3 ± 0.6	0.33
LV ESD (cm)	3.5 ± 0.4	3.9 ± 0.4	0.21	3.6 ± 0.4	3.8 ± 0.5	0.23
IVSd (cm)	1.1 ± 0.1	1.0 ± 0.1	0.30	1.1 ± 0.1	1.2 ± 0.2	0.38
LVPWd (cm)	1.1 ± 0.2	1.1 ± 0.2	0.18	1.0 ± 0.1	1.1 ± 0.2	0.29
LV EF (%)	36 ± 2.8	34 ± 3.5	0.44	53 ± 2.9	55 ± 4.3	0.51
LA diameter (cm)	4.4 ± 4.4	4.6 ± 4.2	0.10	4.1 ± 3.9	4.2 ± 5.6	0.12
RA diameter (cm)	3.7 ± 1.4	3.8 ± 1.5	0.23	3.4 ± 1.6	3.5 ± 1.3	0.28
RV diameter (cm)	3.3 ± 1.2	3.5 ± 1.2	0.55	3.1 ± 1.1	3.2 ± 1.3	0.44

LV: left ventricle; EDD: end-diastolic dimension; ESD: end-systolic dimension; IVSd: inter-ventricular septum in diastole; PWd: parietal wall in diastole; EF: ejection fraction; LA: left atrium; RA: right atrium; RV: right ventricle.

**Table 4:** Predictors of depression in HF patients.

Variable	Univariate Predictors OR (95% CI)	*p* Value	Multivariate Predictors OR (95% CI)	*p* Value
	**HF patients**			
Age	1.231(1.051 to 2.401)	0.03	1.362 (0.989 to 3.824)	0.10
Female gender	2.735 (1.337 to 5.595)	0.04	1.562 (0.809 to 4.024)	0.22
NYHA class > II	2.035 (1.437 to 4.595)	0.01	2.103 (1.389 to 4.700)	0.01
AH	1.018 (0.509 to 3.903)	0.61		
CHD	1.141 (0.819 to 3.898)	0.11		
BMI	1.201 (0.909 to 4.108)	0.10		
BMI ≥ 30 kg/m^2^	1.630 (1.207 to 3.803)	0.01	1.890 (1.199 to 3.551)	0.02
Diabetes	1.105 (0.895 to 3.796)	0.09		
Glycemic level ≥ 8.5 mmol/L	2.105 (1.405 to 3.796)	0.001	2.802 (1.709 to 5.077)	0.01
	**HFrEF**			
Age	1.191(1.091 to 3.001)	0.04	1.701 (0.913 to 3.119)	0.11
Female gender	1.135 (0.937 to 2.095)	0.10		
NYHA class > II	1.830 (1.237 to 3.005)	0.007	2.091 (1.613 to 4.009)	0.01
AH	1.098 (0.811 to 3.211)	0.22		
CHD	1.109 (0.709 to 2.898)	0.31		
BMI	1.009 (0.801 to 3.701)	0.18		
BMI ≥ 30kg/m^2^	1.890 (1.331 to 3.908)	0.01	1.911 (1.401 to 4.018)	0.03
Diabetes	1.205 (0.801 to 3.406)	0.21		
Glycemic level ≥ 8.5 mmol/L	1.961 (1.519 to 3.676)	0.02	2.013 (1.519 to 4.101)	0.01
	**HFpEF**			
Age	1.161(1.007 to 1.399)	0.02	1.301(0.977 to 3.019)	0.08
Female gender	1.511 (1.193 to 3.121)	0.04	1.591(1.110 to 3.029)	0.04
NYHA class > II	1.311 (1.107 to 2.901)	0.08		
AH	1.011 (0.307 to 2.101)	0.22		
CHD	1.122 (0.530 to 2.537)	0.61		
BMI	1.129 (0.817 to 3.160)	0.11		
BMI ≥ 30kg/m^2^	2.047 (1.108 to 3.397)	0.02	1.926 (1.502 to 4.011)	0.02
Diabetes	1.118 (0.909 to 2.915)	0.17		
Glycemic level ≥ 8.5 mmol/L	1.808 (1.015 to 3.541)	0.03	1.703 (1.311 to 3101)	0.03

AH: arterial hypertension; BMI: body mass index; CHD: coronary heart disease; DM: diabetes mellitus.

## Data Availability

Not applicable.

## Supplementary Materials

The following are available online at https://www.mdpi.com/article/10.3390/jcm10235663/s1: Figure S1: flow chart of participants; Table S1: demographic and clinical data between HF patients with and without depression; Table S2: laboratory data between HF patients with and without depression; Table S3: echocardiographic data between HF patients with and without depression.
