# Supplementary material for: Obesity and Uncontrolled Diabetes Predict Depression in HF Patients
Source: J Clin Med. 2021 Nov 30;10(23):5663. doi: 10.3390/jcm10235663 (PMC8658509; doi:10.3390/jcm10235663)
Supplement: Supplementary file 1 [file jcm-10-05663-s001.zip › jcm-1441082-supplementary.pdf]

SUPPLEMENTARY DATA:

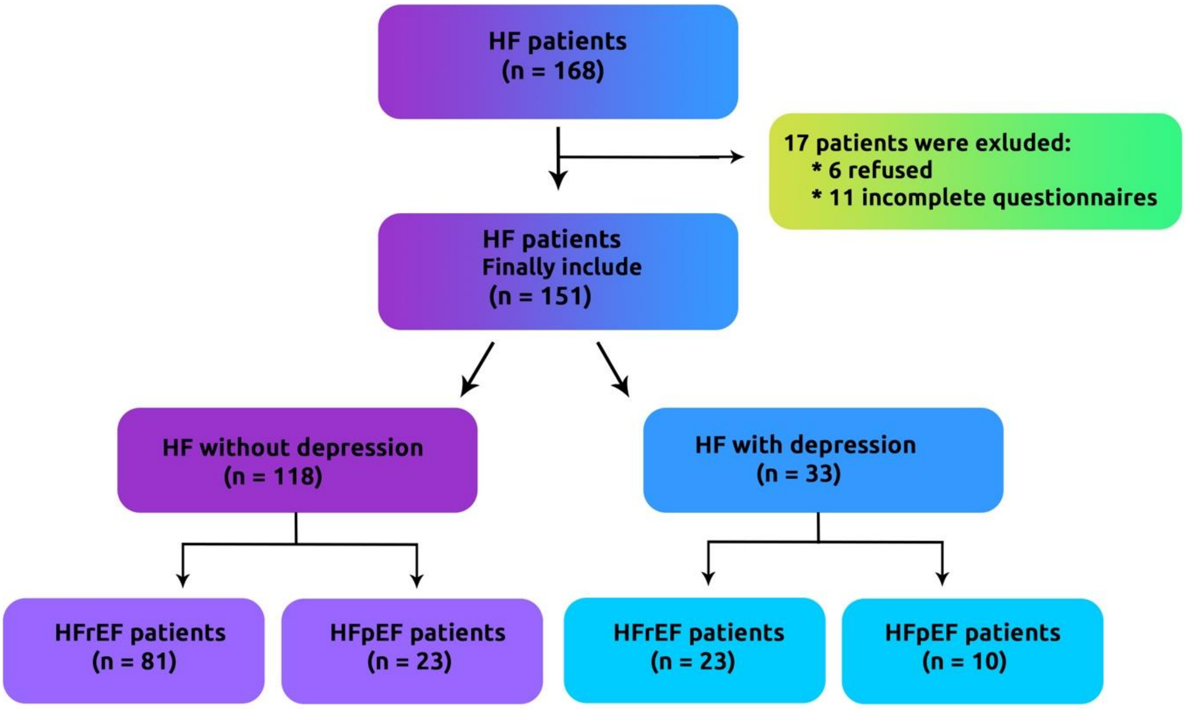

Figure S1. Flow chart of participants

Table S1. Demographic, and clinical data between HF patients with and without depression.

Variable	HF patients (n = 151)	HF without depression (n = 118)	HF with depression (n=33)	P value
Clinical indices				
Age	66.6 ± 11	61.6 ± 11	71.5 ± 10	0.01
Sex (female, %)	79 (52.3)	59 (50)	20 (60.6)	0.02
Smoking (n, %)	29 (19.2)	22 (18.6)	7 (21.2)	0.14
BMI (m/kg^2)	28.2 ± 4.7	27.3 ± 3.8	29.0 ± 5.7	0.04
Underweight (n, %)	1 (0.66)	1 (0.85)	0 (0)	0.09
Normal weight (n, %)	57 (37.8)	42 (35.6)	15 (42.4)	0.03
Overweight (n, %)	59 (39.1)	44 (38.1)	15 (38.6)	0.34
Obese (n, %)	43 (28.5)	31 (26.3)	12 (36.4)	0.02
SBP (mmHg)	107.5 ± 16	110 ± 17	105 ± 14	0.33
DBP (mmHg)	79.5 ± 11	80 ± 12	79 ± 10	0.62
HR (beats/min)	76 ± 14	74 ± 15	78 ± 13	0.21
NYHA class	2.3 ± 1.0	1.9 ± 0.9	2.8 ± 1.1	0.01
Comorbidities				
AH (n, %)	100 (66.2)	80 (67.8)	20 (60.6)	0.10
DM (n, %)	73 (48.4)	55 (46.6)	18 (54.5)	0.02
Dyslipidemia	43 (28.5)	33 (27.9)	10 (30.3)	0.61

CHD (n, %)	66 (43.8)	50 (42.4)	16 (48.4)	0.52
AF (n, %)	71 (47.1)	52 (44.1)	19 (57.6)	0.01
Drugs				
Aspirin (n, %)	118 (78.2)	92 (77.9)	26 (78.8)	0.77
ACE/ARBs (n, %)	125 (82.8)	97 (82.2)	28 (84.8)	0.45
Diuretics (n, %)	107 (70.9)	80 (67.8)	27 (81.8)	0.01
BB (n, %)	111 (73.5)	85 (72.1)	26 (78.8)	0.12
CCB (n, %)	23 (15.2)	18 (15.2)	5 (15.2)	0.88
Antiarrhythmic (n, %)	29 (19.2)	22 (18.6)	7 (21.2)	0.33
Anticoagulation (n, %)	27 (17.9)	19 (16.1)	8 (24.2)	0.04
Statins (n, %)	38 (25.2)	30 (25.4)	8 (24.2)	0.27

AH: arterial hypertension; ACEi: Angiotensin-converting enzyme inhibitors; ARBs: Angiotensin II Receptor Blockers; AF: Atrial fibrillation; BB: Beta blockers; *BMI: Body mass index;* CCB: Calcium channels blockers; *CHD: Coronary heart disease; COPD: Chronic obstructive pulmonary disease; DM: diabetes mellitus; HR: Heart rate; SBD: Systolic blood pressure; DBP: Diastolic blood pressure.*

Table S2. Laboratory data between HF patients with and without depression.

**Variable**	**HF patients (n = 151)**	**HF without depression (n = 118)**	**HF with depression (n=33)**	**P value**
Laboratory				
Glucose (mmol/L)	6.9 ± 2.2	5.6 ± 1.2	8.2 ± 3.2	0.02
Urea (mmol/L)	15.5 ± 5.2	13 ± 4.2	18 ± 6.1	0.004
Creatinine (umol/L)	134 ± 15	122 ± 11	145 ± 19	0.01
Bilirubin (mg/dL)	3.8 ± 1.2	3.5 ± 1.1	4.1 ± 1.3	0.11
ALT (U/L)	31 ± 10	33 ± 11	29 ± 10	0.22
AST (U/L)	38 ± 10	29 ± 10	27 ± 10	0.41
Albumin (g/L)	30.5 ± 8.5	32 ± 8	29 ± 9	0.22
Protein (g/L)	62 ± 11	63 ± 10	60 ± 11	0.14
Cholesterol (mmol/L)	6.7± 3.4	6.5 ± 3.7	6.9 ± 3.1	0.28
Triglyceride (mmol/L)	2.1 ± 0.8	1.9 ± 0.8	2.2 ± 0.9	0.21
WBC (10^3/mm^3)	11.4 ± 4.3	11.2 ± 4.6	11.6 ± 3.2	0.27
RBC (10^6/mm^3)	4.5 ± 1.2	4.8 ± 1.2	4.1 ± 1.1	0.33
Platelet (10^3/mm^3	210± 23	213± 21	208 ± 25	0.11
Iron (umol/L)	9.5 ± 2.8	13 ± 3.1	8 ± 2.5	0.03

ALT; Alanine aminotransferase; AST: Aspartate aminotransferase; RBC: red blood cell; WBC: white blood cell.

Table S3. Echocardiographic date between HF patients with and without depression

**Variable**	**HF patients (n = 151)**	**HF without depression (n = 118)**	**HF with depression (n=33)**	**P value**
LV EDD (cm)	5.1 ± 0.4	5.2 ± 0.4	5.9 ± 0.6	0.03
LV ESD (cm)	3.6 ± 0.4	3.6 ± 0.4	4.3 ± 0.5	0.02
IVSd (cm)	1.15 ± 0.1	1.1 ± 0.1	1.2 ± 0.2	0.40

LVPWd (cm)	1.1 ± 0.2	1.0 ± 0.1	1.1 ± 0.2	0.21
LV EF (%)	47 ± 7.8	55 ± 8.1	39 ± 7.5	0.01
LA diameter (cm)	4.5 ± 4.7	4.1 ± 3.9	4.8 ± 5.6	0.03
RA diameter (cm)	3.5 ± 1.4	3.4 ± 1.6	3.6 ± 1.3	0.13
RV diameter (cm)	3.4 ± 1.2	3.3 ± 1.1	3.5 ± 1.3	0.31

LV: left ventricle; EDD: end-diastolic dimension; ESD: end-systolic dimension; IVSd: inter-ventricular septum in diastole; PWd: parietal wall in diastole; EF: Ejection fraction; LA: left atrial; RA: Right atrium; RV: Right ventricle;
